# Assessment of FAE1 polymorphisms in three Brassica species using EcoTILLING and their association with differences in seed erucic acid contents

**DOI:** 10.1186/1471-2229-10-137

**Published:** 2010-07-01

**Authors:** Nian Wang, Lei Shi, Fang Tian, Huicai Ning, Xiaoming Wu, Yan Long, Jinling Meng

**Affiliations:** 1 National Key Laboratory of Crop Genetic Improvement and National Centre of Plant Gene Research (Wuhan), Huazhong Agricultural University, Wuhan, 430070, China; 2 Plant Breeding Institute, Christian-Albrechts-University of Kiel, Olshausenstrasse 40, D-24098 Kiel, Germany; 3 Oil Crops Research Institute, Chinese Academic of Agriculture Science, Wuhan, 430062, China

## Abstract

**Background:**

*FAE1 *(*fatty acid elongase1*) is the key gene in the control of erucic acid synthesis in seeds of *Brassica *species. Due to oil with low erucic acid (LEA) content is essential for human health and not enough LEA resource could be available, thus new LEA genetic resources are being sought for *Brassica *breeding. EcoTILLING, a powerful genotyping method, can readily be used to identify polymorphisms in *Brassica.*

**Results:**

Seven *B. rapa*, nine *B. oleracea *and 101 *B. napus *accessions were collected for identification of *FAE1 *polymorphisms. Three polymorphisms were detected in the two *FAE1 *paralogues of *B. napus *using EcoTILLING and were found to be strongly associated with differences in the erucic acid contents of seeds. In genomic *FAE1 *sequences obtained from seven *B. rapa *accessions, one SNP in the coding region was deduced to cause loss of gene function. Molecular evolution analysis of *FAE1 *homologues showed that the relationship between the *Brassica *A and C genomes is closer than that between the A/C genomes and *Arabidopsis *genome. Alignment of the coding sequences of these *FAE1 *homologues indicated that 18 *SNPs *differed between the A and C genomes and could be used as genome-specific markers in *Brassica*.

**Conclusion:**

This study showed the applicability of EcoTILLING for detecting gene polymorphisms in *Brassica*. The association between *B. napus FAE1 *polymorphisms and the erucic acid contents of seeds may provide useful guidance for LEA breeding. The discovery of the LEA resource in *B. rapa *can be exploited in *Brasscia *cultivation.

## Background

*Brassica*, which comprises six species, three diploid (*Brassica rapa*, *B. oleracea *and *B. nigra*) and three tetraploid (*B. napus*, *B. juncea *and *B. carianta*), is important in global agriculture and food production. Each of the three diploid species contains one of the original genomes, A, B or C; spontaneous hybridization between two of these three original genomes produced the three tetraploid species [1]. According to recent comparative genomic studies [2-4] the three original genomes arose from the same ancestry, so all six species have complex genomes, especially the three tetraploid plants. In general, three paralogous regions were predicted for the diploids and six for the tetraploids. These *Brassica *species produce an oil that contains essential human dietary components, and some components of the oil are used in industry [5]. During the past 40 years, major traits of *Brassica *have been significantly improved: yield, plant architecture, seed quality and so on. The erucic acid content of the seeds is also a major trait to be considered in improving *Brassica *oilseed. A low erucic acid content is necessary for human health, while a high content is necessary for industrial use [5,6]. Therefore, there is a pressing need to collect and identify different kinds of genetic resources, including high and low seed erucic acid contents, for breeding new *Brassica *cultivars.

*FAE1 *(*fatty acid elongase1*) is the key gene in controlling erucic acid synthesis in *Brassica *seeds. It was originally cloned in *Arabidopsis *by directed transposon tagging with the maize element *Activator *(*Ac*); no intron was found in this gene [7]. The product of *FAE1 *is a condensing enzyme that extends fatty acid chain lengths from C18 to C20 and C22 [8,9]. Many recent reports have addressed the structure of *FAE1 *and its relationship to *Brassica *genomes and seed erucic acid contents. A full cDNA for this gene, as well as the genomic DNA and predicted promoter sequences, are readily available from GenBank. Using this information, full length genomic *FAE1 *sequences were cloned by screening the *B. napus *BAC library with one fragment probe of this gene, and two paralogous *FAE1 *in the genome were found [10]. Two major QTLs were detected using a reference doubled haploid mapping population (TNDH) originating from a Chinese high erucic acid (HEA) cultivar, *B. napus *cv. Ningyou7, and a European low erucic acid (LEA) cultivar, *B. napus *cv. Tapidor. These two QTLs, one located on linkage group/chromosome A8 (A genome) and the other on C3 (C genome), accounted for ~71% of the genetic variation [11]. With developed polymorphic markers and the TNDH mapping population, the two paralogues were regarded as genes under the two QTLs for seed erucic acid contents [10]. Similar findings were reported by Rahman et al. [12]. In 2002 and 2004, one SNP was found in *FAE1 *between a high and a low erucic acid content *B. napus *cultivar [13,14], and this was also reported by Nath et al. [15]. In addition, a four-base nucleotide deletion within the *FAE1 *coding region in an LEA compared to an HEA cultivar was reported. Expression these HEA and LEA *FAE1 *genes in yeast indicated that the SNP or indel causes the differences in erucic acid content [13,14,16]. The *B. rapa *(A) and *B. oleracea *(C) genomes also have paralogous *FAE1 *s [17].

EcoTILLING is a new approach developed from TILLING (Targeting Induced Local Lesions In Genome) and is mainly used to detect SNPs in natural populations. The first successful application of EcoTILLING was to detect variations in several genes in a natural *Arabidopsis *population in 2004 [18]. Many SNPs in a dozen genes were also identified in wild populations of *Populus trichocarpa *[19]. Several reports have indicated that EcoTILLING is a highly efficient approach to detecting genetic variations associated with target traits for crop improvement. Nieto et al. detected one haplotype comprising several grouped SNPs controlling virus susceptibility in a natural melon population. Key SNPs in the resistance gene were also identified and these may be exploited for resistance breeding [20]. However, despite several reports on EcoTILLING, it is relatively difficult to apply the technique to crop improvement, especially for polyploid plants with complex genomes.

Here we report the application of EcoTILLING to the identification of LEA cultivars among natural populations of three *Brassica *species. About 100 accessions of modern cultivars of *B. napus*, seven of *B. rapa *and nine of *B. oleracea *were collected. Variations in the two paralogous, *B. napus FAE1*s, among these cultivars were detected using EcoTILLING. In total, three SNPs/indels were found to be associated with differences in seed erucic acid contents. A new LEA genetic resource was found in the natural *B. rapa *population using sequence alignment. Analysis of the evolutionary relationships and sequence similarities among these three *Brassica *species showed a closer pedigree relationship between the *Brassica *A and C genomes than between the A/C genomes and *Arabidopsis *genome; 18 SNPs were found in the coding region of *FAE1 *and may be used as genome-specific markers to differentiate the A and C genomes.

## Results

### 1. Phenotyping for erucic acid contents of seeds of *Brassica *accessions

One hundred and one accessions of *B. napus *cultivars were collected, mostly modern cultivars. Many of them perform excellently in terms of key traits such as oil content, fatty acid composition, yield and so on. These accessions were derived from different geographic origins: 71 from China, 9 from Sweden, 6 from Germany, 5 from France, 4 from Australia, 1 from Canada, 1 from Denmark, 1 from Poland, and 3 from unknown regions [Additional file 1]. To learn more about the LEA genetic resource, the original LEA ancestors of *B. napus*, 'Liho' and 'Oro', were also collected. Seven accessions of *B. rapa *(5 from China, 1 from Australia and 1 from Finland) (Table 1) and nine of *B. oleracea *(all from China) were also collected (Table 1).

**Table 1:** Accession collection of *B. rapa *and *B. oleracea *accessions for determining erucic acid contents of seeds and their *FAE1 *polymorphisms at coding region

Cultivar No.	Cultivar name	Erucic acid content of seeds (%)	Origin	Positions of nucleotides and amino acid polymorphisms					
***B. rapa***				591^a^	735	898	968	1020	1265
				P197=^b^	A245=	R300=	T323I	K340=	F422=
**1**	Kunshan maquedan	48.58	China	G	C	C	C	G	T
**2**	Sanyue huang	49.76	China	G	C	C	C	G	T
**3**	Hanzhong aiyoucai	45.70	China	G	C	C	C	G	T
**4**	Xinxian huangyoucai	52.02	China	G	C	C	C	G	T
**5**	7801_1	51.26	China	G	C	C	C	G	T
**6**	Jumbuck	1.93	Australia	A	T	C	T	A	T
**7**	Hja 96337	0.90	Finland	A	T	A	T	G	C
***B. oleracea***				489^c^	542	1079	1422	1458	
				Q163=	T181K	F360S	S474=	Y486=	
1	ChunFeng	40.75	China	A	A	T	A	C	
2	Zhonggan11	45.90	China	A	A	T	G	C	
3	Xiaguang Oleracea	43.27	China	G	C	C	G	T	
4	Zhengchun Oleracea	47.63	China	A	A	T	A	C	
5	Jingfeng2	48.20	China	A	C	T	G	Y	
6	Xinfeng	41.40	China	A	A	Y	G	Y	
7	Zaofeng	51.52	China	A	A	Y	A	Y	
8	Improved								
	Niuxin Oleracea	42.07	China	A	A	T	A	C	
9	Hanchun3	41.39	China	G	A	T	G	C	

^a^591 indicates the position 63 bp from the base 'A' in the start codon of *B. rapa FAE1*. P197=^b ^indicates amino acid diversity due to this nucleotide polymorphism. ^c^489 indicates the position 63 bp from the base 'A' in the start codon of *B. oleracea FAE1*. Three haplotypes of *FAE1 *can be inferred among these seven *B. rapa *accessions. Accessions Nos. 1-5 are *B. rapa haplotype *1; accession No. 6 is *B. rapa *haplotype 2 and accession No. 7 is *B. rapa haplotype *3. Seven haplotypes of *FAE1 *can be inferred among these nine *B. oleracea *accessions. Accessions Nos. 1, 4 and 8 are *B. oleracea *haplotype 1; Accessions No. 2, 3, 5, 6, 7 and 9 are *B. oleracea *haplotypes 2 to 7, respectively.

The erucic acid contents of the seeds of the 101 *B. napus *accessions ranged from approximately zero to more than 57% [Additional file 1]. In 60 accessions, the erucic acid contents were between zero and 10%; in one, between 10% and 20%; in eight, between 20% and 30%; in five, between 30% and 40%; and in 27, more than 40%. Thus, the collection comprised *B. napus *pools with low, medium and high erucic acid contents in seeds. In the seven *B. rapa *accessions, two were LEA with almost zero erucic acid contents (Table 1). We found no LEA accession among the nine cultivars of *B. oleracea *(Table 1).

### 2. *FAE1 *polymorphisms in the three *Brassica *species

**Figure 1:**
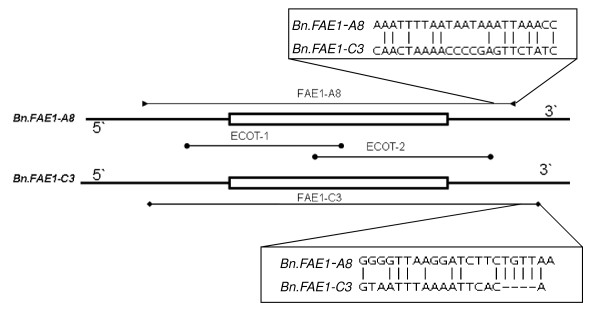
**Regions of the *B. napus FAE1 *genomic sequences amplified by the primer pairs**. Lines represent 5' and 3' genomic sequences of *FAE1 *(~1 kb 5' and 3' for both *Bn.FAE1-A8 *and *Bn.FAE1-C3*) and the rectangles represent the coding region (1-521 bp). FAE1-A8 (line with triangles at the end) indicates the specific primer for *Bn.FAE1-A8*, and FAE1-C3 (line with diamonds at the end) indicates the specific primer for *Bn.FAE1-C3*. ECOT-1 and ECOT-2 (lines with circles at the end) both amplify the *Bn.FAE1-A8 *and *Bn.FAE1-C3 *coding regions (with a short part of the 5' or 3' regions). Sequences alignment for the region of specific primers (reverse primers of FAE1-A8 and FAE1-C3) were showed in corresponding areas.

**Figure 2:**
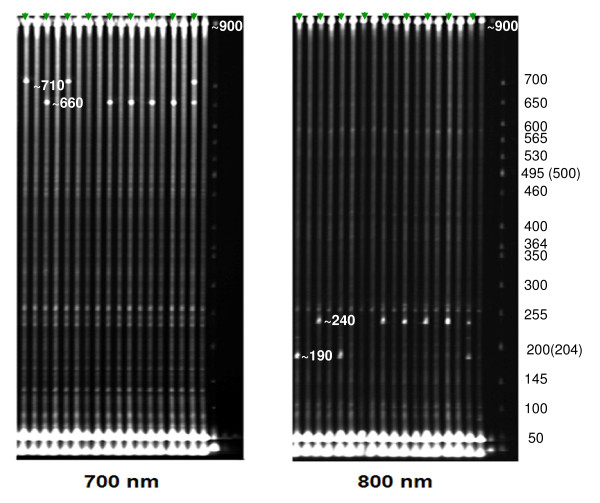
**Detection of polymorphisms for part of *Bn.FAE1-C3 *using EcoTILLING**. DNA from each accession mixed 1:1 with *B. napus *cv. Huayou5 and DNA from each accession alone were used for EcoTILLING, and these two samples were loaded on adjacent lanes on the gel. Lanes with triangle indicate the mixed samples for testing. New bands appeared in both lanes, indicating that the polymorphism was heterozygous. The sizes of DNA ladders and digested PCR products are labelled with corresponding numbers and the unit is bp.

Two paralogous *FAE1 *s were cloned by Wang et al. [10] through screening the *B. napus *BAC library. They were designated as *Bn.FAE1-A8 *and *Bn.FAE1-C3*. Open Reading Frame (ORF) analysis indicated that the coding regions of the two *FAE1 *paralogues were 1521 bp long with no intron and the similarity between them in the HEA *B. napus *cv. Ningyou7 genome was 98.6%. Using primer pairs designed according to these sequences (Table 2, Figure 1) and EcoTILLING, polymorphisms were detected in the 101 *B. napus *accessions. Overall, one position in *Bn.FAE1-A8 *showed polymorphism and two positions in *Bn.FAE1-C3 *showed polymorphisms in the coding regions. Using EcoTILLING, seven accessions were identified individually as heterozygous on *Bn.FAE1-A8 *or *Bn.FAE1-C3*. Figure 2 illustrates the polymorphisms in the second half region of *Bn.FAE1-C3 *with EcoTILLING screening.

**Table 2:** Primer sequences used for PCR amplification

Primer name	Forward primer sequence (5'→3')	Reverse primer sequence (5'→3')
FAE1-A8	GGCACCTTTCATCGGACTAC	GATAGAACTCGGGGTTTTAGTTG
FAE1-C3	GGCACCTTTCATCGGACTAC	TTAACAGAAGATCCTTAACCCC
ECOT1	CGGACCACAAAAGAGGATCC	GTCTCCTTGTTGCACGCAACG
ECOT2	CGTATGCTCTTGTGGTGAGC	GAGAAACATCGTAGCCATC
FAE1-rapa	ATGACGTCCGTTAACGTTAAGC	AAAAGAAACGAAAGAGAGCA
FAE1-oleracea	CTCCGACACACACACTGAGCA	GGGTTTTAGGGTTAAAGATGGTC
M13	CACGACGTTGTAAAACGAC	GGATAACAATTTCACACAGG

To confirm these EcoTILLING results, we randomly sequenced 5-10 samples of each polymorphism and confirmed them at all the positions tested. In total, 19 sequences were obtained; ten of these were selected to confirm the polymorphism on *Bn.FAE1-A8 *(GenBank No.: HM362915 to HM362924); nine were selected to confirm the two polymorphisms on *Bn.FAE1-C3 *(GenBank No.: HM362925 to HM362933, HM362925 and HM362926 contained two polymorphisms). The sequencing results showed that the polymorphic locus of *Bn.FAE1-A8 *was at 845 bp. (We designated the nucleotide 'A' in the start codon of the coding region as position number '1' and the 5' flanking sequence as plus positions; all positions were numbered on the *FAE1 *sequence of the reference sample *B. napus *cv. Huayou5.) In some accessions the position was C; in others it was T [Additional file 1]. Two polymorphic loci of *Bn.FAE1-C *3 were located at 1368-1371 bp (an indel, AGGC deletion) and 1422-1423 bp (an indel, AA deletion). Additional file 1 shows the positions of all the polymorphisms within the HEA *FAE1 *coding region.

Two primer pairs, FAE1-rapa and FAE1-olearacea, were selected to amplify the genomic *FAE1 *sequences of *B. rapa *and *B. oleracea*, respectively (Table 2). Sequencing of the PCR products from each sample of *B. rapa *revealed six polymorphisms in the *FAE1 *coding regions of the seven *B. rapa *accessions (Table 1). Different geographical origins may contribute to sequence differences because five accessions from China had identical sequences and the other two, one from Australia and one from Finland, had different sequences. Five polymorphisms in the coding regions of *FAE1 *were detected from Chinese accessions of *B. oleracea *which showed much greater divergence (Table 1, Additional file 1).

### 3. Association of *FAE1 *polymorphisms with differences in seed erucic acid contents among the three *Brassica *species

According to the EcoTILLING results that identified *FAE1 *polymorphisms in the 101 *B. napus *accessions, two haplotypes of *Bn.FAE1-A8 *and four of *Bn.FAE1-C3 *were inferred. The sequences with nucleotide C and T in the 845 bp position on *Bn.FAE1-A8 *were designated haplotype A8-H0 and A8-H1, respectively. No deletion of *Bn.FAE1-C3 *was designated haplotype C3-H0. A four-base (AGGC) deletion in *Bn.FAE1-C3 *1368-1371 bp and a two-base (AA) deletion in *Bn.FAE1-C3 *1422-1423 bp were designated C3-H1 and C3-H2, respectively. A haplotype with deletions in both positions (1368-1371 bp and 1422-1423 bp) was named C3-H3 (Table 3). The reference accession *B. napus *cv. Huayou5 contained A8-H0 and C3-H0 in its genome and was an HEA cultivar. Furthermore, we found that when the nucleotide changed from C (A8-H0) to T (A8-H1) in *FAE1*, the predicted translated amino acid changed from serine to phenylalanine. Both deletions in *Bn.FAE1-C3 *led to premature stop codon in the predicted ORF.

**Table 3:** Association of differences in seed erucic acid contents with the different *FAE1 *haplotypes of *B. napus*

	C3-H0	C3-H1	C3-H2	C3-H3	LSD test for A8 haplotypes
A8-H0	46.91 ± 4.82^a ^(n = 29^b^)	No plants	30.39 ± 8.53 (n = 3)	No plants	45.36 A
A8-H1	23.71 ± 1.42 (n = 3)	0.67 ± 0.72 (n = 9)	1.02 ± 1.19 (n = 47)	0.55 ± 0.47 (n = 3)	2.04 B
LSD^c ^test for C3 haplotypes	44.73 A^d^	2.78 B	0.673 C	0.55 D	

^a^Erucic acid content of seeds (%)

^b^No. of plants with the two kinds of haplotype in combination

^c^Least significant difference multiple comparisons

^d^Means with different letters following are significantly different (P < 0.01)

The erucic acid content of A8-H0 seeds (mean = 45.36%) was significantly higher than that of A8-H1 (mean = 2.04%) according to ANOVA (GLM model) and an LSD (least significant difference) All-Pairwise Comparison Test (P < 0.01), indicating that C in the 845 bp position of *Bn.FAE1-A8 *is essential for gene function and T may result in loss of function. We found that C3-H0, C3-H1, C3-H2, C3-H3, were also significantly associated with differences in seed erucic acid contents (P < 0.01). The erucic acid content related to C3-H0 (mean = 44.73%) was far higher than that in the other three haplotypes. The erucic acid content of C3-H2 seeds (mean = 0.67%) was significantly lower than that of C3-H1 (mean = 2.78%), showing that the two-base (AA) deletion at 1422-1423 bp in *Bn.FAE1-C3 *inhibited erucic acid synthesis more efficiently that the four-base (AGGC) deletion at 1368-1371 bp. The erucic acid content of haplotype C3-H3 (mean = 0.55%), which harboured both deletions in *Bn.FAE1-C3*, was significantly lower than those of C3-H1 and C3-H2, which harboured one but not both of the deletions. In conclusion, nucleotide C at the 845 bp position of *Bn.FAE1-A8 *is essential for gene function and T might lead to loss of function; the two premature stop codons resulting from the two deletions on *Bn.FAE1-C3 *might also cause loss of function.

Too few *B. rapa *and *B. oleracea *samples were collected for ANOVA to be applied to their data. Alignment of the seven *FAE1 *coding sequences of *B. rapa *showed that three *FAE1 *haplotypes can be inferred. Accession Nos. 1-5 were *B. rapa *haplotype 1, accession No. 6 was haplotype 2 and accession No. 7 haplotype 3 (Table 1). Three of the six polymorphisms were found at positions 591 bp (G/A), 735 bp (C/T) and 968 bp (C/T), and these may be associated with differences in the erucic acid contents of seeds. Plants with nucleotide G at 591 bp and C at 735 bp and 968 bp in *B. rapa FAE1 *had high seed erucic acid contents; those with A at 591 bp and T at 735 bp and 968 bp had low contents. The nucleotide changes at 591 bp and 735 bp did not alter the amino acids predicted from the ORF, but when the C changed to T at 968 bp, the corresponding amino acid changed from threonine to isoleucine. Thus, the difference at position 968 bp in *FAE1 *may have caused the phenotypic difference. Comparing these findings with the nucleotide change in position 845 bp in the A genome of *B. napus*, it may be concluded that the LEA genetic resources in the A genomes in our collection differed between *B. rapa *and *B. napus*.

Alignment of the nine *B. oleracea FAE1 *coding sequences indicated seven haplotypes. Accessions Nos. 1, 4 and 8 were *B. oleracea *haplotype 1; accessions Nos. 2, 3, 5, 6, 7 and 9 were *B. oleracea *2 to 7, respectively (Table 1). Five polymorphisms were found in these nine *FAE1 *coding regions (Table 1). Since no LEA accession was collected for *B. oleracea *in this study, no rule could be found for the genotypes of the HEA accessions of this species.

### 4. Evolutionary relationship of *FAE1 *among the three *Brassica *species

**Figure 3:**
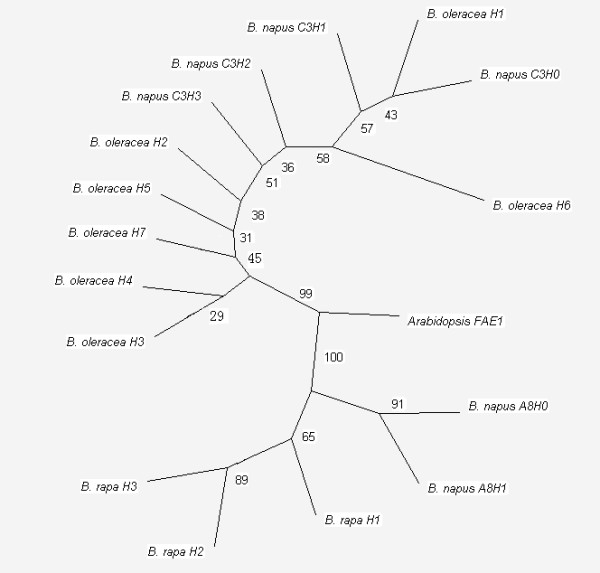
**Phylogenetic tree constructed using *Phylip *3.68 software with neighbour-joining (N-J) algorithm**. A total of 17 *Brassica *sequences - four haplotypes of *Bn.FAE1-C3 *(*B. napus C3H0 *to *C3H3*), two of *Bn.FAE1-A8 *(*B. napus A8H0 *and *A8H1*), three of *B. rapa *(*B. rapa H1 *to *H3*), seven of *B. oleracea *(*B. oleracea H1 *to *H7*), and *Arabidopsis FAE1*- were used for this analysis. These 16 sequences have been submitted to GenBank and their accessions are from GU325717 to GU325732.

In order to investigate the pedigree relationship among the different *Brassica *species and explore the genetic basis of the LEA genetic resource, a phylogenetic tree was constructed using 17 *FAE1 *sequences: six *B. napus FAE1 *haplotypes, seven *B. oleracea *haplotypes, three *B. rapa *haplotyes, and one *Arabidopsis *sequence (Figure 3). This phyogenetic tree indicated that the divergence between the A and C genome *FAE1 *s occurred later than that between *Arabidopsis *and *Brassica *species, which is consistent with other reports [4,21,22]. The tree also showed that the *FAE1 *sequences of the A genome formed two groups, viz. the sequences from *B. napus *and *B. rapa*. However, the C genome revealed more complex groups, making it difficult to draw clear conclusions about the pedigree relationship. In addition, 18 SNPs differed between the A and C genomes, as shown by multiple alignments of 16 *FAE1 *sequences from the three *Brassica *species (Table 4). Analysis of these SNPs showed that many restriction enzymes could distinguish between the A and C genomes. Changes in the nucleotides of 15 of these 18 SNPs led to no changes in the amino acid encoded.

**Table 4:** *FAE1 *polymorphisms between A and C genomes

No.	Positions	Nucleotide Differences Between A and C Genomes	Amino acid difference	Restriction Enzymes
1	63^a^	C T^b^	C21=^c^	
2	72	G A	P24=	AccI, HindII, HpaI, MjaIV, TspGWI
3	168	C T	I56=	BsrDI, BccI, BtgZI
4	312	T C	Y104=	
5	363	G C	S121=	EcoRII, ScrFI, Hpy99I
6	417	T C	T139=	DsaI, SecI
7	531	C T	N177=	AclI, MaeII
8	591	A T	P197=	BccI, BtgZI
9	856	G A	G286R	Eco57MI, EcoRII, GsuI, ScrFI
10	1036	T C	L346=	BseYI, AclI, MaeII
11	1113	T C	D371=	
12	1128	C T	Y376=	
13	1140	T C	D380=	
14	1146	A G	K382=	AluI, Cac8I, CviJI, HindIII
15	1155	T C	I385=	TaqI
16	1184	G A	R395K	
17	1217	C G	A406G	AvrII, HaeIII, SecI, StuI, StyI
18	1464	G C	V488=	MseI, Hin4I

^a^63 indicates the position 63 bp from the base 'A' in the start codon.

^b^The first base is in the A genome and the second is in the C genome.

^c^C21 = indicates no change in the amino acid (synonymous variation)

## Discussion

EcoTILLING, a method developed from TILLING (Targeting Induced Local Lesions in Genome), was successfully applied to *B. napus*, which is considered one of the most complex polyploid plants. We may infer that EcoTILLING is a very powerful method for identifying polymorphisms and for association mapping and developing functional markers for crops. However, gene-specific primer design is the key step for successful application of EcoTILLING to crop improvement. From the first application of TILLING to *Arabidopsis *[23], many studies have detailed the difficulty of developing primers for TILLING and EcoTILLING for crops, because most crops harbour very complex genomes and genomic sequence information is insufficient [10,20,24,25]. Of course, it is relatively easy to design gene-specific primers for plants that have been completely sequenced, such as *Arabidopsis *and rice [18,26]. Two procedures for developing primers for TILLING and EcoTILLING have been reported for crops with complex genomes. First, gene-specific primers can be designed according to sequence differences among multi-copy genes if the relevant genomic information is obtained. Most TILLING and EcoTILLING studies have been carried out using this procedure [18,19,24,25]. Secondly, co-amplified primers can be used for genes that are present in multiple copies and show minor sequence differences among paralogues. Two studies have successfully applied this procedure in TILLING and EcoTILLING [10,27]. In the present study, genomic sequence information was obtained first, and then gene-specific primers were designed for the two paralogues of *FAE1*.

It has been reported that erucic acid is an antinutritional component of seed oil [6]. Therefore, a major objective for rapeseed breeding is to achieve and apply genetic resources with low seed erucic acid contents. During the 1960s, the first LEA *B. napus *germplasm was found in an animal feed rape 'Liho', and then the first *B. napus *LEA cultivar 'Oro' was developed by introducing an LEA genetic resource from 'Liho' [28]. As far as we know, no other LEA genetic resource for breeders has been reported. In this research, we investigated the LEA resources in a collection of 101 modern *B. napus *accessions. The polymorphisms found for *Bn.FAE1-A8 *and *Bn.FAE1-C3 *readily revealed the SNPs/indels associated with differences in seed erucic acid contents. Thus, our results are consistent with other studies showing that SNPs/indels in *FAE1 *corresponded to loss of function of this gene in yeast [13,14,16]. However, comparing these SNPs/indels to the first LEA germplams 'Liho' and 'Oro', we could find only one polymorphisim (1368-1371 bp of *Bn. FAE1-C3*) which may be a new LEA resource. But this deletion was also on the background of the original LEA germplams 'Liho' and 'Oro'. This indicated that no more other LEA resource came out during the past 40 years. Therefore, the LEA genetic resource for *B. napus *is not sufficient and more are required to avoid genetic erosion.

New LEA mutants with a high erucic acid content genetic background were obtained by screening a large *B. napus *EMS mutant population with TILLING [10]. This would be a very efficient method for augmenting LEA genetic resources. In this study, one new LEA genetic resource in *B. rapa *was also found, which differed from that in *B. napus*. There have been many successful reports about the exchange of beneficial traits among six *Brassica *species in the U'Triangle [29-32]. Thus, the LEA genetic resource from *B. rapa *could be introduced to *B. napus *to improve this crop.

The All-Pairwise Comparison Test revealed differences in seed erucic acid contents, related to the four haplotypes of *Bn.FAE1-C3 *in our *B. napus *collection. Both the two-base deletion at 1422-1423 bp and the four-base deletion at 1367-1371 bp affected *Bn.FAE1-C3 *function. The two-base deletion reduced the seed erucic acid content more efficiently than the four-base deletion, and the combination of the two deletions knocked out gene function (Table 3) more effectively than either deletion alone. Thus, to take advantage of these LEA resources, it would be better to introduce both deletions into the target plants so as to breed cultivars with less erucic acid in the seeds.

In the molecular evolutionary analysis of the *FAE1 *sequences of the three *Brassica *species and *Arabidopsis*, we found 18 SNPs that differed between the A and C genomes. Fifteen of these 18 SNPs are silent variations and the original types of *Brassica *species with a full function of *FAE1 *which could produce high erucic acid in seeds. From these results, we may deduce that it is difficult for plants with loss of functional *FAE1 *to survive long in evolution. In other words, plants with higher seed erucic acid contents have a greater capacity to adapt to the environment.

## Conclusions

EcoTILLING has been successfully applied to the identification of *FAE1 *variations in *Brassica*. Polymorphisms in *Bn.FAE1-A8 *and *Bn.FAE1-C3 *were strongly associated with differences in the erucic acid contents of seeds. Different nucleotide deletions in *Bn. FAE1-C3 *reduced the erucic acid content to different degrees. The discovery of a new LEA resource enlarged the pool of genetic resources and could be used for LEA breeding.

## Methods

### 1. Plant materials

Seeds of most of the *B. napus *and *B. rapa *accessions were obtained from the Chinese Crop Germplasms Information System (CGRIS, a germplasm repository for collecting worldwide genetic resource of oilseed crop) and seeds of the two LEA ancestor cultivars, 'Liho' and 'Oro', were obtained from the Australian Temperate Field Crops Collection (ATFCC). Seeds of the nine accessions of *B. oleracea *were obtained from the Horticulture Department of Huazhong Agricultural University (HAU). The plants were grown in the field and young leaves were picked to extract DNA with a JYZ-3-1-2 isolation kit (Genebase Gene-Tech Co. Ltd, Shanghai, China). Self-pollinated seeds from *B. napus *and *B. oleracea *cultivars were harvested for the determination of erucic acid content. Owing to the self-incompatibility of *B. rapa*, sib-crossings between two plants with uniform genetic backgrounds were done for each accession collected, and seeds were then harvested.

### 2. Erucic acid contents of seeds

The erucic acid contents of self-pollinated seeds were determined by gas chromatography (GC) as described in [33] with little modification. First, 30-50 oven-dried seeds were crushed and transferred to 50 ml screw-capped centrifuge tubes. Thereafter, 1 ml ether/Sherwood oil reagent (1 ether: 1 Sherwood oil, by volume) and 1 ml methanol reagent (23 g potassium hydroxide in 1000 ml methanol) were added. The mixture was incubated at room temperature (25°C) for at least 40 min then 20-30 ml distilled water was added to the tubes. Finally 0.4-0.8 μl upper layer was loaded on to the GC instrument (HP 6890 series). Erucic acid content was determined by measuring the area of the peak.

### 3. Screening of *B. napus FAE1 *polymorphisms by EcoTILLING

Two complete paralogous *FAE1 *genomic sequences, EU543282 (*Bn.FAE1-A8*) and EU543283 (*Bn.FAE1-C3*), were retrieved from NCBI. Because these two paralogues are very similar, it was difficult to design gene-specific primer pairs for the coding region. Therefore, two primer pairs, FAE1-A8 and FAE1-C3, were designed with 5' and 3' flanking sequences of the two paralogous genes (Table 2, Figure 1). The two forward primers corresponding to the 5' flanking sequence were the same (their position in genes is -167 to -147 bp), whereas the reverse primers corresponding to the 3' flanking sequence differed between *Bn.FAE1-A8 *and *Bn.FAE1-C3 *(1711 to 1734 bp for *Bn.FAE1-A8*, 1728 to 1750 bp for *Bn.FAE1-C3*, Figure 1)*. *PCR products amplified with the two primer pairs were both about 1.9 kb and were unsuitable for EcoTILLING because 1.5 kb is the longest size for the detection instrument [34]. Another two primer pairs, designated ECOT-1 and ECOT-2, (Table 2), were designed according to the two paralogous coding regions, and were used to amplify the first half fragment (-74 to 1025) and the second half fragment (727-1589) of *Bn.FAE1-A8 *or *Bn.FAE1-C3*, respectively (Figure 1). Sequence of universal primers M13F was added to 5' ends of ECOT-1F and ECOT-2F as adaptors; and M13R was also added to ECOT-1R and ECOT-2R (Table 2). The universal primers M13F and M13R were labelled at the 5' end with IRD 700 and IRD 800, separately (MWG Biotech, Inc., Ebersberg, Germany). FAE1-A8 and FAE1-C3 were used to amplify the two paralogous genes separately in the first PCR reaction, and then these PCR products were amplified by nested PCR reaction. This reaction contained a mixture of gene-specific primers with M13 tails and the universal labelled M13 primers.

DNA samples were isolated from each *B. napus *accession (single plant, self-pollinated seeds harvested from this plant were used for erucic acid content test) and 50 ng DNA was used for 10 μl PCR reaction. *B. napus *cv. Huayou5 is a very old cultivar in China and it was speculated to have high divergence to the modern *B. napus *cultivars according to experiences of breeders. Aiming to detect more polymorphic sites on EcoTILLING gel, a replicate DNA sample of this accession was used as reference in this study. To test whether the two paralogues were heterozygous, these DNA samples were mixed 1:1 with the reference sample and also individually. The products of the first PCR which amplified by gene-specific primer pairs FAE1-A8 or FAE1-C3 were diluted 50-fold to carry out the nested PCR reaction. The screening protocol followed that described by Till et al [34]. CEL1 enzyme was extracted from celery according to Oleykowski et al [35]. To avoid PCR errors, all PCR reactions were amplified with proof-reading *pfu taq *polymerase (Tiangen Biotech, Inc., Beijing, China).

To confirm the polymorphisms identified by EcoTILLING, the accessions that showed polymorphism on a gel were randomly selected for amplification with the primer pairs FAE1-A8 or FAE1-C3 again with proof-reading *pfu taq *polymerase. The PCR products were electrophoresed on agarose gels to recover the target bands, and the purified products were sequenced with ABI 3730. In the analysis of the sequences obtained, chromatograms of each sample were checked to reduce PCR or sequencing errors.

### 4. Identify of *FAE1 *polymorphisms in *B. rapa *and *B.oleracea *

Three *B. rapa *sequences, AF400050, Y14975 and Y14974, and two *B. oleracea *sequences, Y14981 and AF05440, were retrieved from NCBI. For these sequences, which contained *Bn.FAE1-A8 *and *Bn.FAE1-C3*, a total of five primer pairs were designed to amplify the complete *B. rapa *and *B. oleracea *genomic *FAE1 *sequences (Table 2). Because of their high efficiency, FAE1-rapa and FAE1-oleracea were selected to amplify the seven *B. rapa *and nine *B. oleracea *accessions. The PCR products were electrophoresed on agarose gels to recover the target bands, and the purified products were sequenced with ABI 3730. Sequences of these cultivars were aligned using ClustalX 1.83 (downloaded from NCBI), and the polymorphisms were also determined by comparing them with their sequence traces to avoid negative SNPs.

### 5. Data analysis and phylogenetic tree construction

Associations between *FAE1 *polymorphisms of *B. napus *and seed erucic acid contents were analyzed by ANOVA (GLM model) and an LSD (Least Significant Difference) multi-comparison test (SAS 8.2 software).

To construct the phylogenetic tree for *FAE1*, six types of *FAE1 *sequences of *B. napus *(A8-H0, A8-H1, C3-H0, C3-H1, C3-H2 and C3-H3), three of *B. rapa *(*B. rapa *haplotypes 1 to 3), seven of *B. oleracea *(*B. oleracea *haplotypes 1 to 7) and the *FAE1 *sequence of *Arabidopsis *(AT4G34520) were collected, and phylogenetic and molecular evolutionary analyzes were conducted using Phylip 3.68 with a neighbour-joining (N-J) algorithm http://evolution.genetics.washington.edu/phylip.html[36]. To analyze genome-specific markers for the A and C genome, the above 17 sequences except the *FAE1 *sequence of *Arabidopsis *were aligned with ClustalX 1.83 with default parameters [37], and SNPs that differed between the A and C genomes were analyzed using the PARSESNP (Project Aligned Related Sequences and Evaluate SNPs) web tool for identifying restriction enzymes and predicting amino acid changes [38].

## Authors' contributions

NW, FT and HN screened the polymorphisms with EcoTILLING and carried out all the sequencing validation and data analysis. FT extracted DNA of some of the 101 *B. napus *and determined the erucic acid contents of the seeds by GC for *B. napus *and *B. oleracea*. XW planted all these accessions of *Brassica *and provided the data about the erucic acid contents of *B. rapa *seeds. NW and LS wrote the paper and participated in editing it. YL, XW and JM edited the paper; JM and LS also supported the project. All authors read and approved the final manuscript.

## Supplementary Material

Additional file 1 **Collection of *B. napus *for determining seed erucic acid contents and their polymorphisms in *Bn.FAE1-A8 *and *Bn.FAE1-C3***. Click here for file
