# Supplementary material for: Assessment of FAE1 polymorphisms in three Brassica species using EcoTILLING and their association with differences in seed erucic acid contents
Source: BMC Plant Biol. 2010 Jul 1;10:137. doi: 10.1186/1471-2229-10-137 (PMC3017795; doi:10.1186/1471-2229-10-137)
Supplement: Additional file 1 — Collection of B. napus for determining seed erucic acid contents and their polymorphisms in Bn.FAE1-A8 and Bn.FAE1-C3. [file 1471-2229-10-137-S1.DOC]

**Additional file 1.** accessions of *B. napus* for determination of erucic acid content in seeds and polymorphisms on *Bn.FAE1-A8* and *Bn.FAE1-C3*.

| **No.** | **Cultivar**  **Name** | **Erucic acid**  **content**  **in seeds (%)** | **Origin**^a^ | ***Bn.FAE1-A8***  **(845** ^b^**)** | ***Bn.FAE1-C3***  **(1368-1371)** | ***Bn.FAE1-C3***  **(1422-1423)** |
| --- | --- | --- | --- | --- | --- | --- |
| **1** | Huyou16 | 0.70 | China | T | Deletion^c^ | Deletion |
| **2** | HAU_1 | 23.25 | China | T | AGGC | AA |
| **3** | 2A165 | 1.26 | China | T | AGGC | Deletion |
| **4** | HAU_2 | 23.51 | China | C | AGGC | Deletion |
| **5** | Huashuang3 | 0.26 | China | T | Deletion | AA |
| **6** | Huashuang4 | 0.36 | China | T | AGGC | Deletion |
| **7** | Huashuang5 | 0.54 | China | T | AGGC | Deletion |
| **8** | Yuyou2 | 0.26 | China | T | Deletion | AA |
| **9** | Yuyou5 | 0.01 | China | T | AGGC | Deletion |
| **10** | Huyou15 | 0.69 | China | T | AGGC | Deletion |
| **11** | Huyou17 | 0.17 | China | T | AGGC | Deletion |
| **12** | Huyou18 | 0.87 | China | T | AGGC | Deletion |
| **13** | Shilifeng | 2.41 | China | T | Deletion | AA |
| **14** | Ningyou10 | 0.60 | China | T | AGGC | Deletion |
| **15** | Ningyou12 | 0.28 | China | T | AGGC | Deletion |
| **16** | Ningyou14 | 0.04 | China | T | AGGC | Deletion |
| **17** | Ningyou16 | 0.06 | China | T | AGGC | Deletion |
| **18** | Yangyou6 | 0.49 | China | T | AGGC | Deletion |
| **19** | Suyou3 | 0.02 | China | T | Deletion | Deletion |
| **20** | Suyou5 | 0.06 | China | T | AGGC | Deletion |
| **21** | Zheshuang72 | 0.02 | China | T | Deletion (H)^d^ | Deletion (H) |
| **22** | Zhongshuang4 | 0.18 | China | T | AGGC | Deletion |
| **23** | Zhongshuang5 | 0.35 | China | T | AGGC | Deletion |
| **24** | Zhongshuang6 | 1.49 | China | T | AGGC | Deletion |
| **25** | Zhongshuang7 | 0.02 | China | T | AGGC | Deletion |
| **26** | Zhongshuang8 | 31.93 | China | T (H) | Deletion | AA |
| **27** | Zhongshuang9 | 0.08 | China | T | Deletion | AA |
| **28** | Zhongshuang10 | 2.25 | China | T | AGGC | Deletion |
| **29** | Qingyou14 | 22.45 | China | T(H) | AGGC | Deletion |
| **30** | Longyou2 | 0.5 | China | T | AGGC | Deletion |
| **31** | Xiangyou13 | 41.93 | China | C | AGGC | AA |
| **32** | Xiangyou17 | 38.49 | China | C | AGGC | AA |
| **33** | LvyouX | 0.50 | China | T | Deletion | AA |
| **34** | Bullet | 0.87 | Sweden | T | AGGC | Deletion |
| **35** | Grouse | 0.14 | Australia | T | AGGC | Deletion |
| **36** | Mnty | 1.12 | Australia | T | Deletion | AA |
| **37** | HAU_3 | 39.94 | China | C | AGGC | Deletion |
| **38** | Jingzhoubianyi | 41.27 | China | C | AGGC | Deletion (H) |
| **39** | Chuanyou18 | 19.92 | China | T(H) | AGGC | Deletion |
| **40** | Zhongyou821 | 43.05 | China | C | AGGC | AA |
| **41** | Altex | 1.92 | Sweden | T | AGGC | Deletion |
| **42** | B104 | 27.57 | China | T | Deletion (H) | AA |
| **43** | Baihua | 21.22 | China | T | AGGC | Deletion (H) |
| **44** | Ceres | 0.40 | German | T | Deletion | AA |
| **45** | Chuanyou11 | 43.49 | China | C | AGGC | AA |
| **46** | Cresor | 2.16 | France | T | AGGC | Deletion |
| **47** | Doral | 0.59 | German | T | AGGC | Deletion |
| **48** | Erra | 0.51 | German | T | AGGC | Deletion |
| **49** | Ganyou11 | 49.29 | China | C | AGGC | AA |
| **50** | Global | 0.25 | Sweden | T | Deletion | AA |
| **51** | Huayou11 | 48.09 | China | C | AGGC | AA |
| **52** | Huayou13 | 48.38 | China | C | AGGC | AA |
| **53** | Huayou14 | 47.23 | China | C | AGGC | AA |
| **54** | Huayou16 | 40.90 | China | C | AGGC | AA |
| **55** | Huayou5 | 47.55 | China | C | AGGC | AA |
| **56** | Huayou6 | 45.16 | China | C | AGGC | AA |
| **57** | Huayou9 | 47.48 | China | C | AGGC | AA |
| **58** | Huyou9 | 39.65 | China | C | AGGC | AA |
| **59** | SV.Juno | 0.19 | Sweden | T | AGGC | Deletion |
| **60** | Jupiter | 0.50 | Sweden | T | AGGC | Deletion |
| **61** | Ledos | 0.01 | German | T | AGGC | Deletion |
| **62** | Major | 27.74 | France | C | AGGC | Deletion |
| **63** | Nestor | 2.61 | Sweden | T | AGGC | Deletion |
| **64** | Optima | 2.23 | Danmark | T | AGGC | Deletion |
| **65** | Primor | 0.93 | France | T | Deletion | Deletion |
| **66** | Chuxianbaihua | 1.71 | China | T | AGGC | Deletion |
| **67** | Xianglongyou2 | 48.72 | China | C | AGGC | AA |
| **68** | Xianglongyou3 | 43.9 | China | C | AGGC | AA |
| **69** | Xinghuanghua | 50.69 | China | C | AGGC | AA |
| **70** | Youguangye | 22.58 | China | T | AGGC | AA |
| **71** | Youyan2 | 46.87 | China | C | AGGC | AA |
| **72** | Zheyou7 | 49.97 | China | C | AGGC | AA |
| **73** | Qingyou2 | 48.77 | China | C | AGGC | AA |
| **74** | Quinta | 1.73 | German | T | AGGC | Deletion |
| **75** | Bronowski | 2.08 | Poland | T | AGGC | Deletion |
| **76** | Ganyou3 | 50.40 | China | C | AGGC | AA |
| **77** | Niklas | 0.73 | Sweden | T | AGGC | Deletion |
| **78** | Huayou12 | 53.07 | China | C | AGGC | AA |
| **79** | Andor | 0.89 | Sweden | T | AGGC | Deletion |
| **80** | Bienvenu | 1.90 | France | T | AGGC | Deletion |
| **81** | Brink | 0.78 | Sweden | T | AGGC | Deletion |
| **82** | Xianglongyou1 | 36.60 | China | C | AGGC | AA |
| **83** | Tribute | 1.24 | Canada | T | AGGC | Deletion |
| **84** | ORO | 0.59 | Australia | T | AGGC | Deletion |
| **85** | Ningyou7 | 45.89 | China | C | AGGC | AA |
| **86** | Tapidor | 0.67 | France | T | AGGC | Deletion |
| **87** | LIHO | 6.92 | German | T | AGGC | Deletion |
| **88** | Yiyou3 | 57.91 | China | C | AGGC | AA |
| **89** | Yunyou7 | 56.53 | China | C | AGGC | AA |
| **90** | 88-4-43-1 | 44.58 | China | C | AGGC | AA |
| **91** | Huayou5 | 0.19 | China | T | AGGC | Deletion |
| **92** | Xiangyou15 | 0.10 | China | T | AGGC | Deletion |
| **93** | Karoo | 0.25 | Australia | T | AGGC | Deletion |
| **94** | Huashuang2 | 0.78 | China | T | Deletion | AA |
| **95** | Vanguard | 1.52 | unknown | T | AGGC | Deletion |
| **96** | Chengdu186 | 25.31 | China | T | AGGC | AA |
| **97** | HAU_4 | 48.70 | China | C | AGGC | AA |
| **98** | Chuan91 | 49.13 | China | C | AGGC | AA |
| **99** | Marctic | 1.24 | unknown | T | AGGC | Deletion |
| **100** | HAU_5 | 47.87 | China | C | AGGC | AA |
| **101** | Comet | 3.24 | unknown | T | AGGC | Deletion |

^a^Indicates accessions come from these countries according to the Chinese Crop Germplasms Information System.

^b^845 indicates the position at the 845bp from the base ‘A’ in start code on the ORF of reference sample *B. napus cv.* Huayou5.

^c^There is a deletion in this locus on the genomic sequence comparing to the reference sample *B. napus cv.* Huayou5.

^d^H indicates this polymorphism is heterozygote.
